# Long-Term Mechanical Properties, Drying Shrinkage, and Creep Behaviour of Manufactured-Sand Concrete in Plateau Regions: 1-Year Measurements and Analysis

**DOI:** 10.3390/ma19153228

**Published:** 2026-07-29

**Authors:** Yuanjie Liang, Xia Li, Gang Ma

**Affiliations:** 1 School of Emergency Management, Chongqing Vocational Institute of Safety Technology, Chongqing 404100, China; yuanjieliang2024@163.com (Y.L.); liang_keyan@163.com (X.L.); 2 School of Civil Engineering, Central South University, Changsha 410083, China

**Keywords:** plateau regions, manufactured-sand concrete, mechanical properties, drying shrinkage, creep behaviour, pore structure

## Abstract

**Highlights:**

- A 1-year measurement on the long-term performance of manufactured-sand concrete in plateau regions was conducted.
- Plateau harsh environment deteriorates the performance of manufactured sand.
- Synergistic effect of silica fume and calcium sulfate whiskers enhances the performance of manufactured-sand concrete.
- Plateau harsh environment increases pore volume ranging from 20 to 200 nm.

**Abstract:**

Infrastructure construction in plateau areas not only needs to take into account the impact of harsh climatic conditions, but also faces the problem of raw material shortage. Herein, this work investigates the long-term mechanical properties, drying shrinkage, and creep behaviour of manufactured-sand concrete in plateau regions via 1-year measurements. Results show that the plateau harsh environment coarsens the pore structure of manufactured-sand concrete, leading to the 365-day compressive strength and elastic modulus dropping by at most 10.2% and 5.3%, respectively, while the 365-day drying shrinkage and specific creep increased by at most 15.3% and 9.4%, respectively. Meanwhile, with the synergistic effect of silica fume, calcium sulfate whiskers and shrinkage-reducing agent, the 365-day compressive strength increased by 12.3%, and drying shrinkage and specific creep were reduced by 10.5% and 16.6%, respectively, resulting from its dense microstructure effect. Overall, this work offers guidance for preparing high-performance concrete in plateau areas, promotes the resource utilisation of manufactured sand, and has significant implications for enhancing the service life of concrete while reducing construction costs.

## 1. Introduction

With the vigorous advancement of infrastructure construction in the western high-altitude regions of China, the adaptability of cement-based materials in the harsh environment of high-altitude areas has attracted much attention [1,2,3]. The western plateau features variable temperatures, dryness, and strong winds, which pose significant challenges to the performance of cement-based materials [4,5,6]. Furthermore, the fragile ecological environment and difficult transportation in high-altitude areas lead to a shortage of raw materials; for instance, the scarcity of river sand resources has necessitated the use of manufactured sand [7,8]. Therefore, the performance of manufactured-sand concrete—specifically its long-term mechanical properties, drying shrinkage, and creep behaviour—under the harsh conditions of the plateau warrants investigation, as it holds significant value for infrastructure construction in these regions.

In 2024, the total output of sand and gravel in China was 15.2 billion tons, and the natural sand resources are gradually becoming scarce at present, resulting from excessive extraction and long-term consumption [9,10,11]. Against this background, manufactured sand with diverse raw material sources has gradually begun to dominate the sand market. Manufactured sand and river sand differ in terms of particle shape and stone powder content, resulting in significant variations in the mechanical properties, volume stability, and durability of concrete [12,13]. This has attracted the attention of many scholars; for example, Huang et al. [14] pointed out that manufactured-sand concrete has a denser interfacial transition zone (ITZ) structure due to the rough particle morphology of manufactured sand. Shen et al. [15] found that there is no significant difference in the mechanical properties between manufactured-sand concrete and river sand concrete with the same gradation. In addition, previous results have indicated that stone powder is the most crucial factor affecting the strength and durability of manufactured-sand concrete [16]. Ding et al. [17] reported that stone powder content is positively correlated with long-term compressive strength when the stone powder content is less than 13%. As for the drying shrinkage and creep behaviour, Li et al. [18] reported that the uniaxial creep modulus of manufactured-sand concrete was lower than that of river sand (RS), with a difference exceeding 11%. Liu et al. [19] found that carbonation curing can reduce the drying shrinkage of manufactured-sand concrete, with the reduction rate exceeding 35.5%, resulting from CaCO_3_ optimising pore structure and inhibiting water evaporation as well as capillary contraction. While many studies have examined the application of manufactured sand, the varying production processes and raw material sources limit the generalizability of previous conclusions, and further research is particularly needed for the plateau harsh environments.

The annual temperature variation in plateau areas is relatively small, while the daily variation is relatively large, and the annual average day–night temperature difference can reach 15 °C [20,21]. It also has distinct climatic characteristics such as low-pressure, dryness, strong winds and intense solar radiation, which significantly affect the long-term performance of concrete materials [22,23]. Ma et al. [24] reported that low pressure can reduce the compressive strength of mortar and increase its drying shrinkage, which relates to the weakened hydration process and the increased total pore volume. Zeng et al. [25] indicated that day–night temperature difference harms the macro-mechanical properties, pore structure and micro-morphology of cement paste, resulting from the coupling effect of thermal deformation and thermal fatigue. Cagnon et al. [26] found that a low-humidity environment accelerates the moisture migration within the cementitious materials, thereby increasing drying shrinkage. In summary, the current research on the mechanical and drying shrinkage properties of cement-based materials in high-altitude environments has been extensive. However, most of the current studies are conducted in laboratory-scale conditions and lack validation in real field environments.

At present, the adverse effects of the harsh environment on cement-based materials have drawn the attention of many scholars, and many improvement measures have also been continuously attempted [27,28,29]. For example, exploring the utilisation of shrinkage agent to prevent drying shrinkage, as reported by Qin et al. [30], shrinkage agent can reduce the pore solution surface tension to inhibit water migration, thereby minimising the drying shrinkage of cement-based materials in high-altitude, low-humidity and windy environments. Jin et al. [31] found that fly ash and limestone powder enhanced hydration via their nucleation effect and refined pore structure, thereby improving mortar performance and mitigating the negative impacts of low atmospheric pressure. Bai et al. [32] discovered that nano-SiO_2_ can reduce the curing time needed for strength and durability development, thereby lessening the impact of low temperatures on the durability of cement-based materials. These explorations have provided a reference for improving the performance of manufactured-sand concrete in high-altitude areas.

Although many studies have examined the harsh environments effects—such as low-pressure air and significant temperature variations—on concrete performance, research in actual high-altitude complex environments is rare [33,34]. Additionally, given the current shortage of raw materials in these areas, investigating the performance of manufactured-sand concrete under long-term exposure in high-altitude conditions is crucial, which holds significant potential for enhancing concrete durability and reducing construction costs. Here, to promote the application of manufactured sand in high-altitude areas, the long-term performance of manufactured-sand concrete was investigated in Xizang, China. In detail, the compressive strength, elastic modulus, drying shrinkage, creep and related pore structure of manufactured-sand concrete over a period of one year were measured. On the whole, this work contributes to the design and construction of infrastructure in high-altitude areas.

## 2. Materials and Methods

### 2.1. Raw Materials

**Figure 1:**
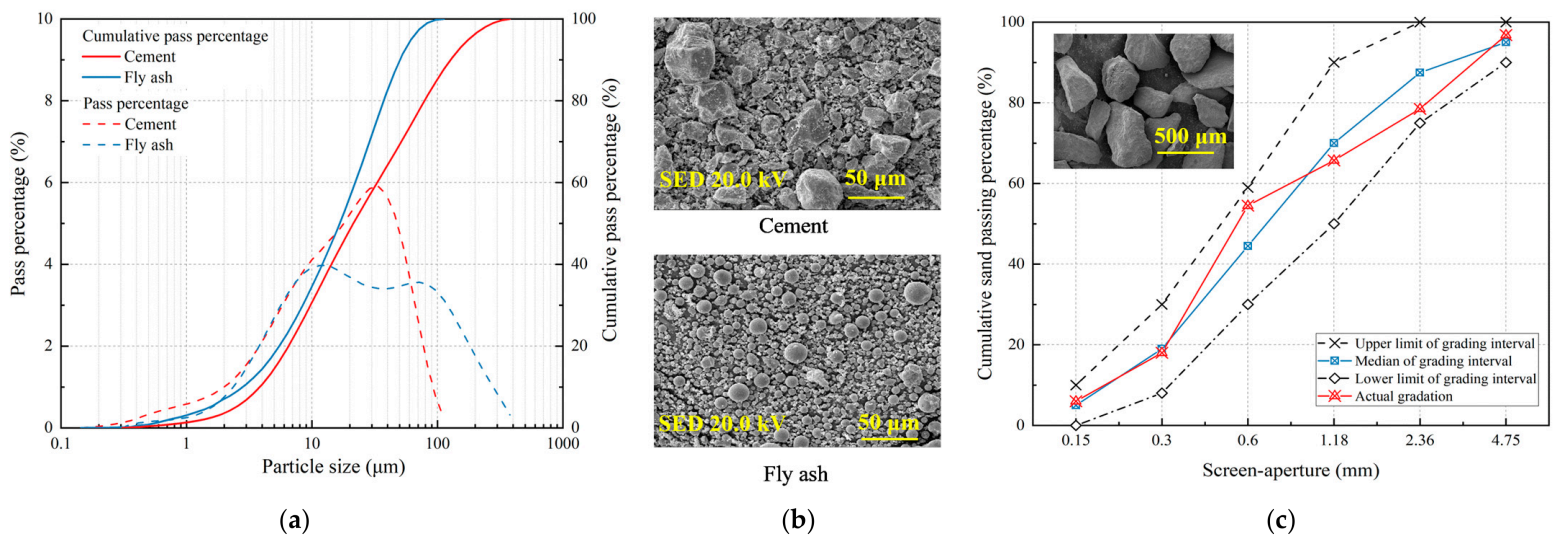
Particle size distribution and morphology of raw materials, (**a**) particle size distribution, (**b**) micro-morphology of cement and fly ash observed by SEM, and (**c**) the grading curve and particle morphology of the investigated manufactured sand.

In this work, the P.O 42.5 ordinary cement produced by Xizang Gaozheng Building Materials Co., Ltd. (Lhasa, China) was employed, and its apparent density and specific surface area are 3.11 g/cm^3^ and 358 m^2^/kg, respectively, obtained by the liquid replacement method and nitrogen adsorption test. Fly ash with an apparent density of 2.18 g/cm^3^ and a specific surface area of 390 m^2^/kg from Lingwu, Ningxia, was utilised as a supplementary cementitious material. In addition, silica fume with an apparent density of 2.1 g/cm^3^ and a specific surface area of 20,600 m^2^/kg, along with calcium sulphate whiskers having an apparent density of 2.71 g/cm^3^, an average length of approximately 30 μm, and an aspect ratio of 18, were employed to enhance concrete microstructure. Table 1 shows the chemical composition of the cementitious materials analysed by X-ray fluorescence spectrometry, while Figure 1a illustrates the particle size distribution and Figure 1b shows the micro-morphology, which were obtained by laser particle size analysis and scanning electron microscope (SEM) tests, respectively.

Manufactured sand, produced by the local sand factory in Xizang, was used as fine aggregate, and its powder content, fineness modulus and apparent density are 8.2%, 2.89, and 2740 kg/m^3^, respectively, obtained by the sand washing method, sieving method, and liquid displacement method. Figure 1c shows the grading curve and particle morphology. Meanwhile, crushed stone with a particle size ranging from 5 to 19.5 mm was utilised as coarse aggregate. In addition, superplasticiser and shrinkage-reducing agents were also employed as chemical admixtures.

### 2.2. Specimen Preparation and Test Site

According to the on-site construction mix proportion, two different mix proportions were utilised to prepare the C55 concrete specimen. In detail, #MSC_S is the on-site construction mix proportion, and #MSC_M denotes the modified mix proportion incorporating silica fume and calcium sulphate whiskers, where the proportions of silica fume and whiskers are 2% and 1% (wt %), respectively, of the cementitious materials. Silica fume is selected for its ability to significantly enhance the microstructure of concrete through both physical filling and chemical reactions. Its ultra-fine particles, characterised by a large specific surface area, effectively fill the gaps between cement particles, thereby reducing porosity [35]. Additionally, these particles react with calcium hydroxide generated during cement hydration to form calcium silicate hydrate (C-S-H) gel, resulting in a denser structure. Conversely, calcium sulfate whiskers are chosen for their exceptional strength and toughness, providing dual benefits of reinforcement and filling due to their sub-nanometre fibre characteristics [36,37]. In addition, the shrinkage-reducing agent was employed instead of superplasticizer to reduce the surface tension of the pore solution, and the specific ratio is listed in Table 2.

**Figure 2:**
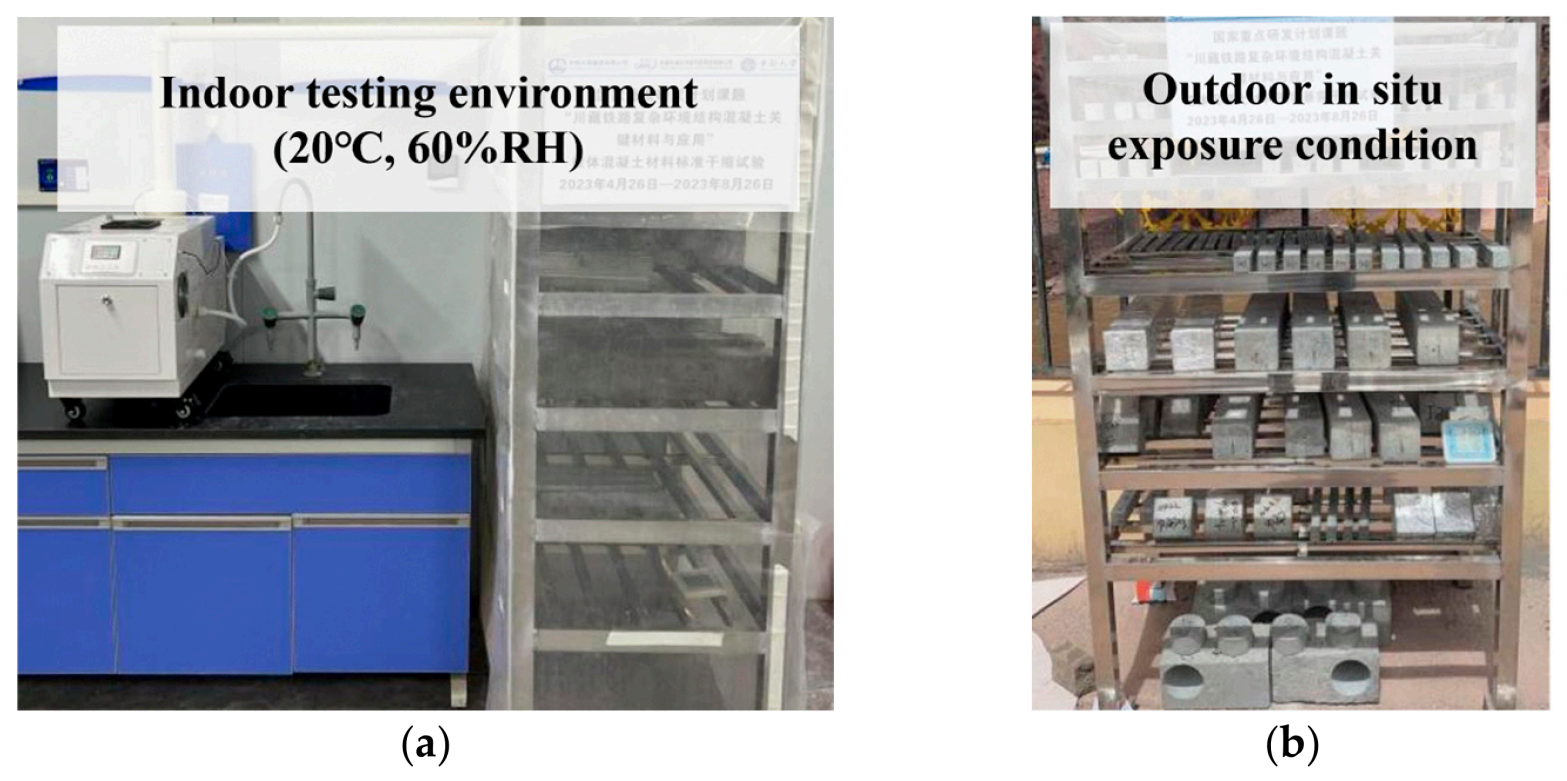
Photos of two different experimental environments, (**a**) indoor testing environment, and (**b**) outdoor in situ exposure condition.

The raw materials were first added to the mixer and dry-stirred for 90 s before water and chemical admixtures were added, followed by an additional 180 s of stirring [38]. The concrete mixture was then poured into moulds and vibrated evenly on a vibration table. The specimens were covered with cling film and allowed to stand for 24 h before removing the moulds. Among them, the cubic specimens with a side of 100 mm were prepared for the compressive strength test, and those with a size of 100 × 100 × 300 mm^3^ were prepared for the elastic modulus test, as well as the drying shrinkage test. The creep test employed cylindrical specimens with a diameter of 50 mm and a height of 150 mm, and cylindrical specimens with a diameter of 100 mm and a height of 50 mm were also prepared for capillary water absorption and electric flow tests. They were then placed in a standard curing room (20 °C, 98% RH) for 2 days, after which they were moved to an indoor testing environment (20 °C, 60% RH) and an outdoor exposure area for testing, as displayed in Figure 2. It should be noted here that creep specimens were cured in the standard curing room for 14 days.

It should be pointed out here that the location of the test site is approximately 3800 m in Xizang, China (32°52′ N, 97°06′ E). Monitored environmental parameters indicate that this site has distinct characteristics of large temperature differences, strong wind and dryness, and intense radiation. In detail, the annual average radiation intensity was 105.5 × 10^3^ μW/cm^2^, the annual average temperature was 7.8 °C, the daily maximum temperature difference was 21.4 °C, and the extreme maximum and minimum temperatures were 33.4 °C and −20.7 °C, respectively. The relative humidity ranges from 10.6% to 65.0%, and the maximum wind speed is 11.5 m/s.

### 2.3. Test Methods

**Compressive strength test:** It was conducted on 100 mm in-side cubic specimens at ages of 14, 28, 90, 180 and 365 days, with a loading rate of 0.8 MPa/s, and the average value of three specimens was treated as the final result.

**Elastic modulus test:** The axial compressive strength of 100 × 100 × 300 mm^3^ concrete specimens was first tested at ages of 14, 28, 90, and 365 days, and then three-cycle loading-unloading was carried out according to the standards, with an upper limit of 30% and a lower limit of 0.5% axial compressive strength. The elastic modulus *E* can be obtained from the deformation after three cycles, which can be expressed as

(1) $$E=\frac{F_{30\%}-F_{0.5\%}}{A}\times \frac{L}{\Delta L}$$

where *A* represents the cross-sectional area of the specimen, that is, 100 × 100 mm. *L* and △*L* denote the initial height of the specimen and the deformation induced by the three cycles of loading, respectively. *F*_30%_ and *F*_0.5%_ mean the 30% and 0.5% of axial compressive strength, respectively.

**Figure 3:**
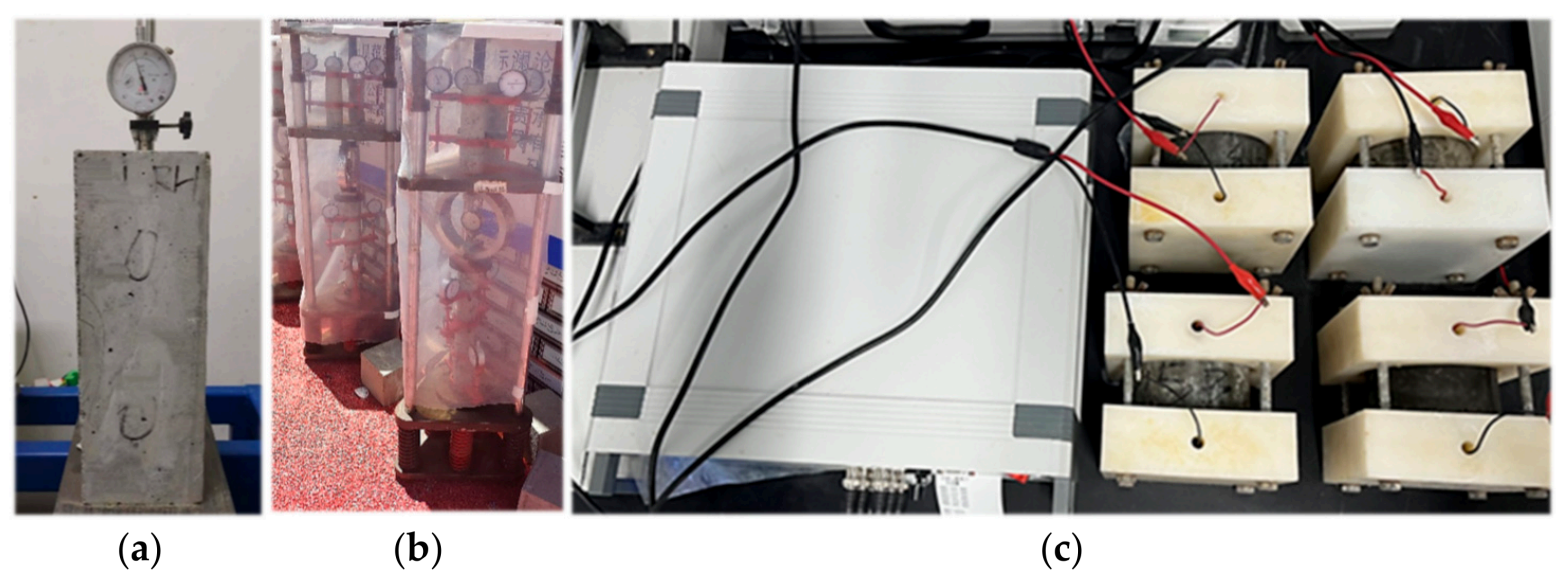
Photos of test equipment, (**a**) drying shrinkage test, (**b**) creep test, and (**c**) electric flow test.

**Drying shrinkage test:** After being cured in the standard curing room for 3 days, the 100 × 100 × 300 mm specimens were moved to the indoor testing environment (20 °C, 60%RH) and the outdoor exposure area. The specially designed concrete shrinkage frame and micrometres were employed to test drying shrinkage, as presented in Figure 3a, and thus the drying shrinkage value can be calculated via Equation (2).

(2) $$\varepsilon =\frac{L_{0}-L_{t}}{300}\times 10^{6}$$

where *ε* represents the drying shrinkage value. *L*_0_ and *L*_t_ denote the reading of the micrometre at the initial time and test age *t*, respectively.

**Creep test:** After being cured in the standard curing room for 14 days, the cylindrical specimens with a diameter of 50 mm and a height of 150 mm were employed to conduct creep tests in the outdoor exposure area, as presented in Figure 3b. The axial compressive strength of cylindrical specimens was determined first to determine the applied creep stress, that is, 30% of the axial compressive strength of cylindrical specimen [11,39]. The strain of specimens under sustained load within one year was recorded, and at the same time, the drying shrinkage under the same conditions was monitored. On this basis, the creep strain can be obtained.

**Capillary water absorption test**: The cylindrical specimen with a diameter of 100 mm and a height of 50 mm was employed to carry out capillary water absorption test at age 28 days. In detail, the specimen was placed in an oven to dry until it reached a constant weight first, and subsequently, the side of specimen was sealed with silicone rubber coating. The specimen was then immersed in tap water to a depth of 5 ± 1 mm, and the specimen mass at different times was recorded. The capillary water absorption can be reflected by the capillary water absorption height, written as

(3) $$I=\frac{m_{t}-m_{0}}{S\times \rho_{\omega }}\times 10^{3}$$

where *I* represents the seepage height. *m*_0_ and *m*_t_ denote the mass at the initial and test time *t*, *S* is the cross-sectional area of the specimen, and *ρ*_w_ is the water density.

**Electric flow test:** The cylindrical specimen with a diameter of 100 mm and a height of 50 mm was employed to carry out the electric flow test at age 28 days, and the pre-treatment step was the same as that of capillary water absorption test. A 3% NaCl solution was injected into the cathode test tank, and 0.3 mol/L NaOH solution was injected into the anode test tank. The 6 h electric flow was measured via a concrete chloride ion electric flow measurement instrument, as shown in Figure 3c.

**Pore structure test:** Mercury intrusion porosimetry (MIP) method was employed to analyse the pore diameters of concrete specimen. Crushed specimens weighing 1 g were immersed in isopropanol to halt the hydration process and then dried in a vacuum oven [40]. Subsequently, pore size distribution was measured using the AutoPore IV 9500 fully automatic mercury porosimetry instrument from Micromeritics Corporation, Norcross, GA, USA.

## 3. Results and Analysis

### 3.1. Compressive Strength and Elastic Modulus

**Figure 4:**
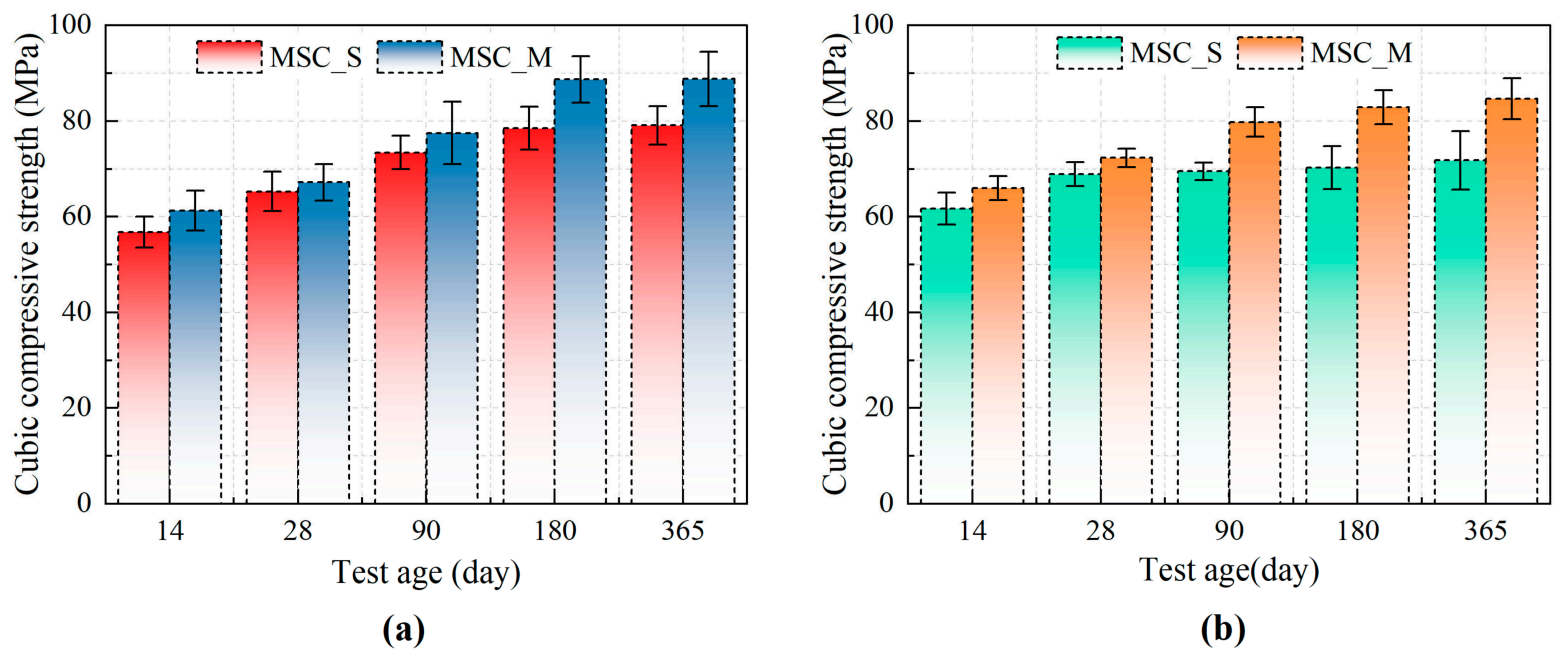
Compressive strength of manufactured-sand concrete within one year, (**a**) indoor testing environment, and (**b**) outdoor in situ exposure condition.

**Figure 5:**
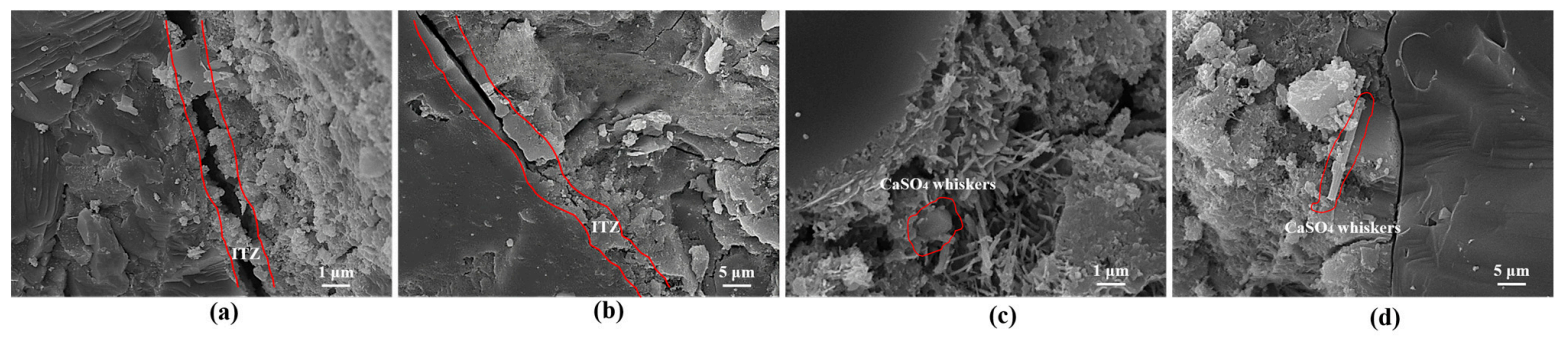
SEM observation of manufactured-sand concrete at 28 days, (**a**,**b**) MSC_S concrete under indoor testing environment and outdoor in situ exposure condition, and (**c**,**d**) MSC_M concrete under indoor testing environment and outdoor in situ exposure condition.

Figure 4 shows the compressive strength of manufactured-sand concrete under indoor testing and outdoor in situ exposure conditions within one year. Regardless of whether under indoor testing or outdoor in situ exposure conditions, the compressive strength of both mix proportions gradually increases with age. The 14-day and 28-day compressive strength of concrete under outdoor in situ exposure conditions was higher than those under indoor testing conditions, while it presents an opposite pattern after 180 days. The 28-day compressive strength of MSC_S and MSC_M specimens under outdoor in situ exposure conditions was 5.5% and 7.6% higher than that under indoor testing conditions. This relates to the fact that direct sunlight outdoors causes the specimen temperature to rise, which accelerates the hydration process and results in higher compressive strength at an early age [41,42]. The 365-day compressive strength of MSC_S and MSC_M specimens under outdoor in situ exposure conditions was 10.2% and 4.8% lower than that under indoor testing conditions, indicating that the harsh environment of plateaus deteriorates the long-term mechanical properties. The alternating temperatures, low humidity in high-altitude areas accelerated the deterioration of pore structure, as seen in Figure 5a,b, resulting in a slow or stagnant increase in long-term mechanical properties, which has been proved by Wang et al. [43]. This relates to how the harsh plateau environment can widen the interface transition zone (ITZ) and pore structure, causing stress concentration and crack formation, which weakens the overall mechanical strength and durability of cement-based materials.

Compared with the MSC_S specimen, the MSC_M specimen, which incorporates silica fume and calcium sulphate whiskers, has a higher compressive strength under both indoor testing and outdoor in situ exposure conditions. In detail, under indoor testing conditions, the 14-day, 90-day, and 365-day increase amounts were 7.9%, 5.6%, and 12.3%, respectively, while those were 6.9%, 14.8% and 17.9%, respectively, under outdoor in situ exposure conditions. This confirms that the synergistic effect of silica fume and calcium sulfate whiskers can significantly increase the compressive strength of manufactured-sand concrete, especially in complex environments at high altitudes, as seen in Figure 4b. This relates to the physical filling and chemical volcanic ash reaction of silica fume, and the bridging effect of calcium sulfate whiskers [36,44], as shown in Figure 5c,d. It can be observed that calcium sulfate whiskers is surrounded by a large amount of hydrated products such as calcium aluminosilicate, suggesting that adding calcium sulfate whiskers results in the transformation of ITZ from a loose and disordered state to a compact microstructure.

**Figure 6:**
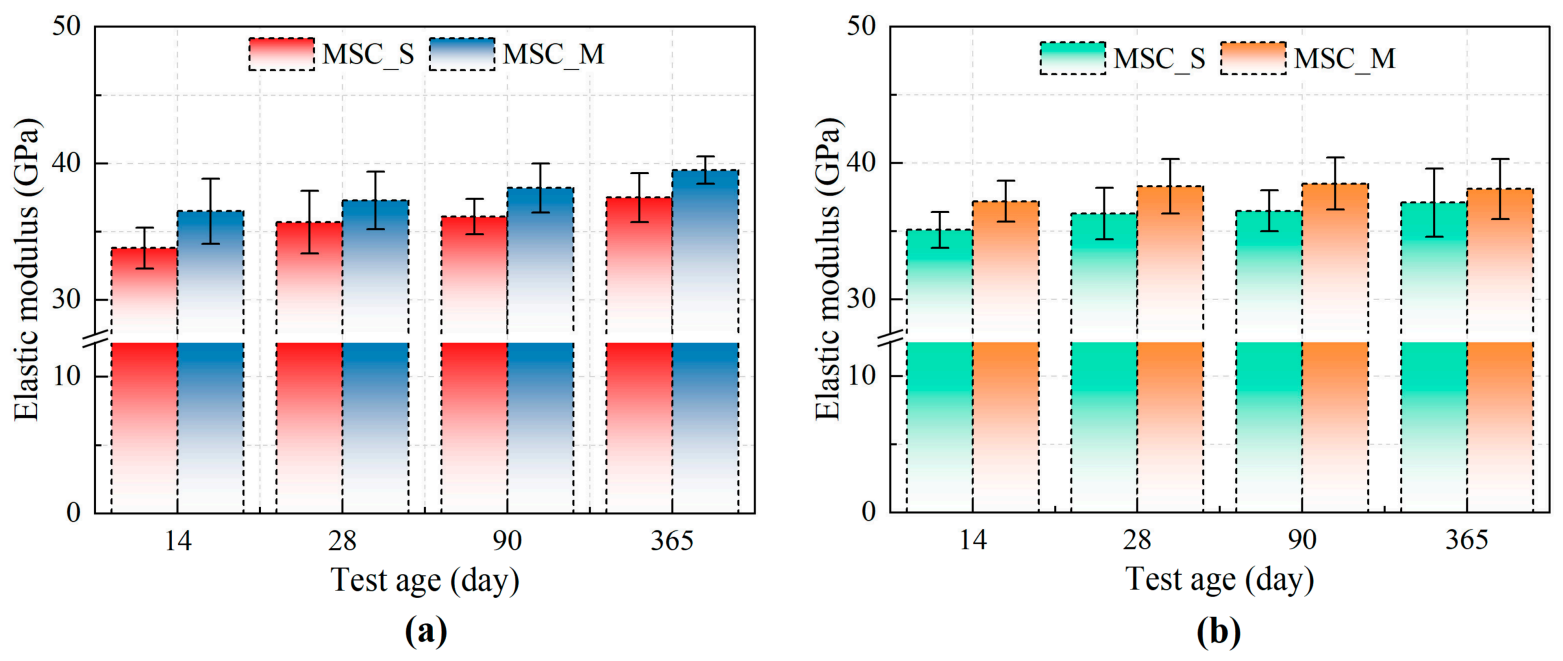
Elastic modulus of manufactured-sand concrete within one year, (**a**) indoor testing environment, and (**b**) outdoor in situ exposure condition.

Figure 6 shows the elastic modulus of manufactured-sand concrete under indoor testing and outdoor in situ exposure conditions within one year. It can be observed that the 14-day, 28-day, 90-day, 365-day elastic modulus of MSC_S specimen under indoor testing condition was 7.9%, 4.5%, 5.8%, and 5.3%, respectively, lower than those of MSC_M specimen, as seen in Figure 6a, and the variation range under outdoor in situ exposure condition was 5.9%, 5.5%, 5.4%, and 2.7%, as seen in Figure 6b. This shows that the co-effect of silica fume and calcium sulfate whiskers enhanced the elastic modulus of manufactured-sand concrete, but this positive effect can be weakened by the complex environment of high-altitude areas.

### 3.2. Drying Shrinkage

**Figure 7:**
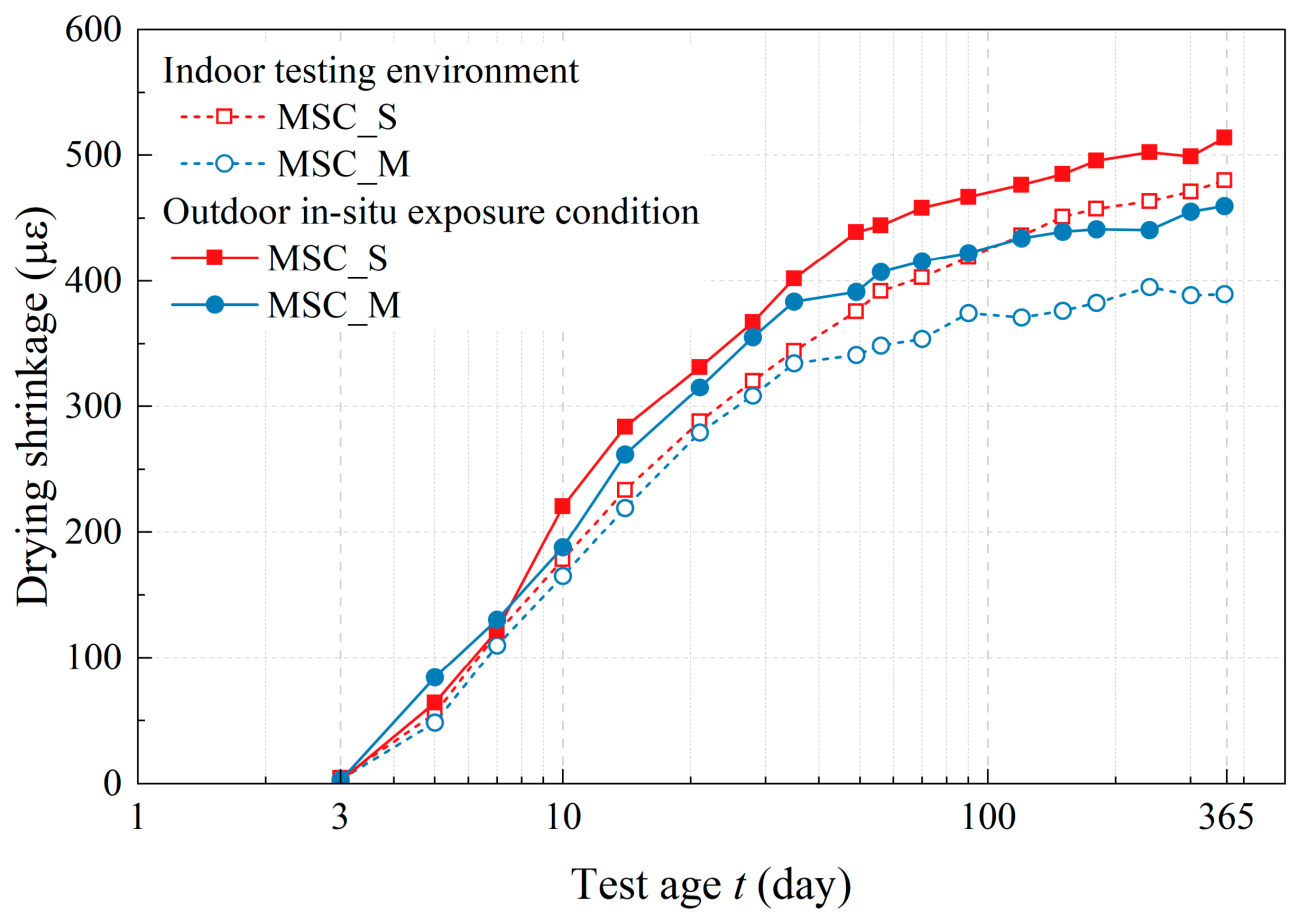
Drying shrinkage of manufactured-sand concrete within one year.

Figure 7 shows the drying shrinkage of MSC_S and MSC_M specimens under indoor testing and outdoor in situ exposure conditions. In detail, in the 3–28-day age, the drying shrinkage strain of manufactured-sand concrete developed rapidly, and 28-day drying shrinkage was approximately 70% of that at 180 days. The growth rate of drying shrinkage gradually slowed down and stabilised within 28–180 days, and only minor changes occurred within 180–365 days.

The drying shrinkage of MSC_S and MSC_M specimens was much greater than that in the indoor testing conditions. The 180-day drying shrinkage of MSC_S under outdoor in situ exposure conditions was 497 με, which is 9.7% higher than that in the indoor testing conditions, and that at 365 days was 7.0%. For the MSC_M specimens, the variation range at 180 days and 365 days was 15.3% and 18%, respectively. This is related to the harsh environment in plateau areas, such as large temperature differences and low humidity, which significantly accelerates the internal moisture migration in manufactured-sand concrete and further aggravates its drying shrinkage [42].

Furthermore, by comparing the drying shrinkage of MSC_S and MSC_M specimens, it can be observed that the drying shrinkage of the MSC_M specimens was less than that of the MSC_S specimens in both indoor and outdoor testing conditions. In detail, the 180-day and 365-day drying shrinkage of MSC_S under indoor testing conditions were 16.4% and 18.9% more than that of the MSC_M specimen, and that under outdoor testing conditions were 10.9% and 10.5%, respectively. On one hand, the addition of silica fume and calcium sulfate whiskers has made the microstructure denser, reducing the pathways for water migration [44]. On the other hand, the MSC_M specimen was also incorporated with shrinkage-reducing agent, and thus, the viscosity of pore solution increases, restricting the evaporation of pore water. Meanwhile, the addition of shrinkage-reducing agent led to a decrease in the pore solution tension, and the drying shrinkage was reduced under the action of capillary tension [45]. In addition, the variation in the outdoor testing conditions was smaller than that in the indoor testing conditions, suggesting that although the plateau harsh environment inevitably deteriorates the performance of manufactured-sand concrete, the added modifying materials, i.e., silica fume, calcium sulfate whiskers, and shrinkage-reducing agent inhibitors, still effectively prevent drying shrinkage deformation.

### 3.3. Creep Behaviour

**Figure 8:**
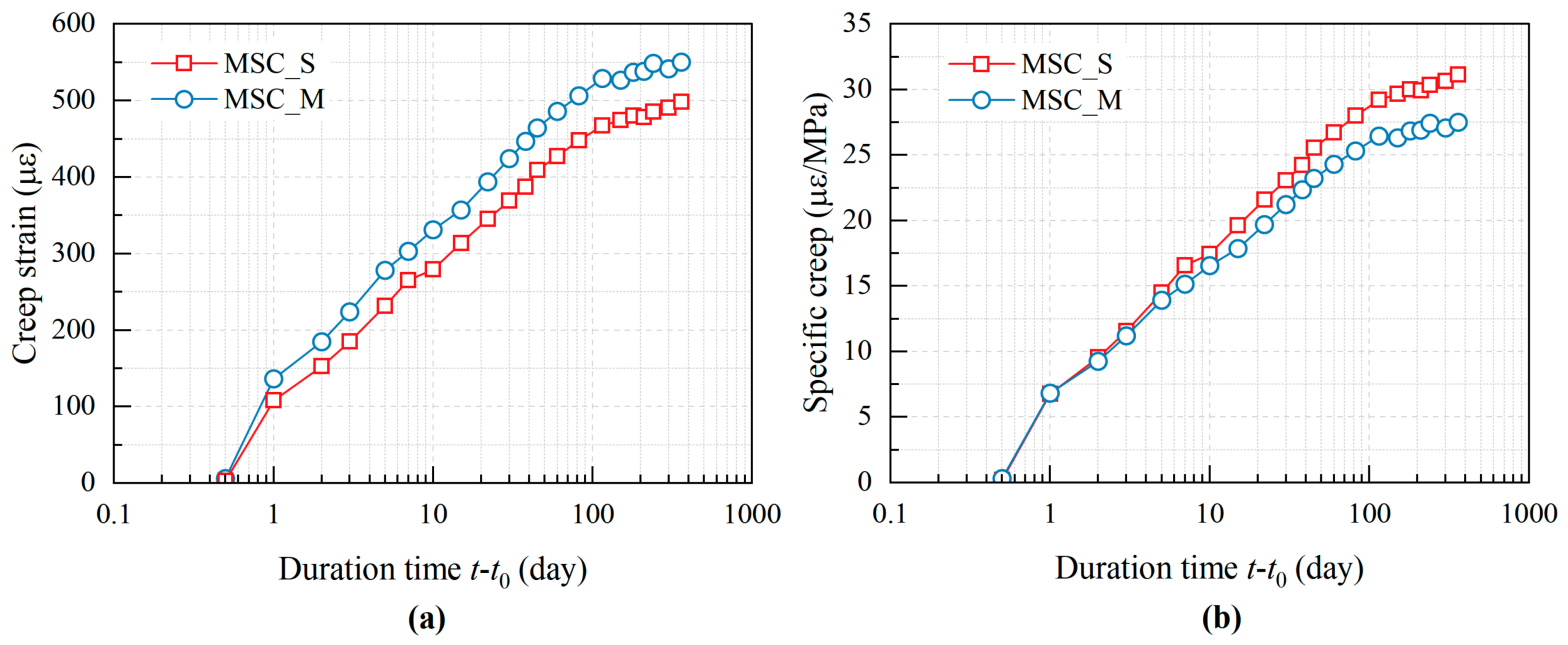
Creep properties of manufactured-sand concrete within one year under in situ exposure conditions, (**a**) creep strain, and (**b**) specific creep.

It is necessary to measure the axial compressive strength of cylindrical concrete specimens to determine the creep load. In this work, the 14-day axial compressive strength of MSC_S and MSC_M specimens was 54.2 MPa and 67.8 MPa, respectively, and thus, the creep load for MSC_S and MSC_M specimens was 16 MPa and 20 MPa, respectively. On this basis, the creep test was conducted under outdoor in situ exposure conditions, and the creep strain was obtained, as presented in Figure 8a.

It can be observed that the creep strain development of MSC_S and MSC_M specimens in situ exposure conditions can be divided into three stages, that is, 0–14 days, 14–90 days, and 90–365 days. In 0–14 days, the creep strain developed rapidly, and the 14-day creep strain of MSC_S and MSC_M specimens was 314 με and 357.3 με, respectively, accounting for 63.1% and 64.9%, respectively, of the observed 365-day creep. In the 14–90-day period, the creep growth rate slowed, and the 90-day creep strain for the MSC_S and MSC_M specimens reached 457.2 με and 518.9 με, respectively, indicating increases of 45.6% and 45.2% compared to the 14-day creep strain. After 90 days, the creep strain increased sharply and then decreased, and the 365-day creep strain for the MSC_S and MSC_M specimens was 498.3 με and 550.2 με, respectively, which were just 9.0% and 6.0% higher than the 90-day creep strain. This suggests that in the plateau harsh environment, the creep strain of manufactured-sand concrete mainly occurs within 90 days, with the 14-day creep strain accounting for more than 63%.

Overall, the creep strain of the MSC_M specimen was greater than that of MSC_S specimen, as seen in Figure 8a. However, this does not mean that the addition of silica fume and calcium sulfate whiskers has increased the creep deformation of manufactured-sand concrete. Instead, the applied creep load of 20 MPa was greater than that of the MSC_S specimen, i.e., 16 MPa, resulting in a larger creep strain of MSC_M specimen. Therefore, the specific creep of MSC_S and MSC_M specimens was calculated, as shown in Figure 8b. It can be observed that the difference between the two specimens was minimal during the first 7 days, and subsequently it gradually increased. In detail, the 90-day specific creep of MSC_S and MSC_M specimens was 28.6 με/MPa and 25.9 με/MPa, and that at 365 days was 31.1 με/MPa and 27.5 με/MPa. Compared with the MSC_S specimen, the 90-day and 365-day specific creep of the MSC_M specimen has dropped by 9.4% and 11.6%, which confirms that silica fume and calcium sulfate whiskers have an inhibitory effect on the creep behaviour of manufactured-sand concrete. As mentioned earlier, the synergy of two materials leads to an increase in the elastic modulus, which means an enhancement in its resistance to deformation [37].

### 3.4. Anti-Penetrability Properties

**Figure 9:**
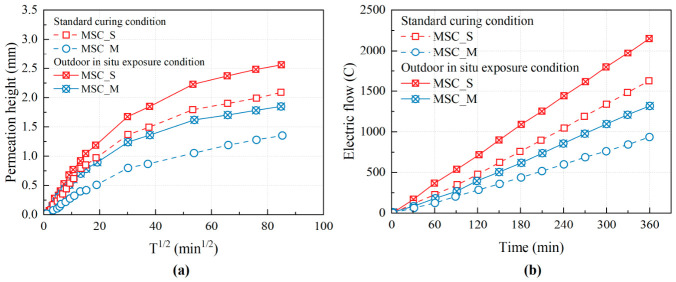
Anti-penetrability properties of manufactured-sand concrete, (**a**) capillary water absorption, and (**b**) electric flow.

Figure 9a shows the 28-day capillary water absorption of MSC_S and MSC_M specimens under indoor testing and outdoor in situ exposure conditions. The absorption height of manufactured-sand concrete increased significantly during the initial 0–15 h, while the increase rate gradually slowed down and then stabilised in 15–120 h. This is because water gradually entered the smaller-sized pores over time, leading to an increased resistance to capillary water absorption. Compared with the specimen under indoor testing conditions, the specimen under outdoor in situ exposure conditions has a higher absorption height, with the 28-day absorption height of the MSC_S and MSC_M specimens under outdoor in situ exposure conditions being 2.6 mm and 1.9 mm, respectively, 24% and 36% higher than those under indoor testing conditions. This indicates that exposure to high-altitude environments resulted in the deterioration of the pore structure.

Additionally, the 28-day absorption height of MSC_M specimens was lower than that of MSC_S specimens, showing a 33% difference under indoor testing conditions and a 27% difference under outdoor in situ exposure conditions, as shown in Figure 9a. This further demonstrates that the addition of silica fume and calcium sulfate whiskers can fill the interconnected pores within manufactured-sand concrete, resulting in a more significant improvement in the permeability property.

The 28-day electric flow of MSC_S and MSC_M specimens was also analysed, as displayed in Figure 9b, and the electric flow of manufactured-sand concrete under indoor testing conditions was lower than that under outdoor in situ exposure conditions. In detail, the electric flow of the MSC_S and MSC_M specimens under indoor testing conditions was 32% and 42%, respectively, lower than that under outdoor in situ exposure conditions. This relates to the accelerated water evaporation driven by the plateau harsh environment, and there are more coarse capillary interconnecting pores, making chloride ions migrate more easily. Meanwhile, the specimen, which incorporates silica fume and calcium sulfate whiskers, has a smaller electric flow, with a 42% difference under indoor testing conditions and a 33% difference under outdoor in situ exposure conditions, see Figure 9b. This indicates that the combination of silica fume with calcium sulfate whiskers and the modification with shrinkage-reducing agent can significantly enhance the chloride ion permeability resistance of manufactured-sand concrete in high-altitude areas.

### 3.5. Pore Structure

**Figure 10:**
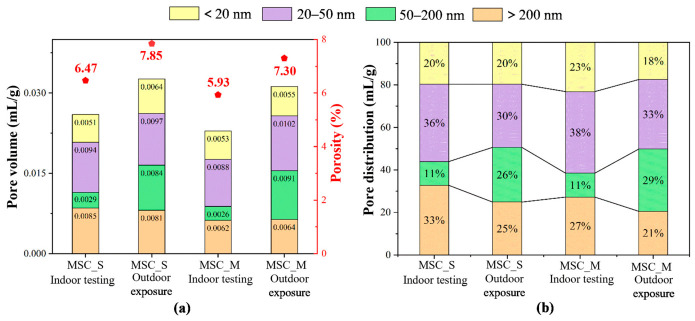
The 180-day pore structure of manufactured-sand concrete analysed by the MIP method, (**a**) pore volume distribution and porosity, and (**b**) proportion of pore distribution.

Pore structure of manufactured-sand concrete of MSC_S and MSC_M specimens under different test conditions was investigated by the MIP method to analyse the adverse effects of the plateau harsh environment on the macroscopic performance of manufactured-sand concrete, as well as the modification mechanisms of adding silica fume, whiskers and shrinkage-reducing agent. In detail, the plateau harsh environment increased the porosity of manufactured-sand concrete, and the 180-day porosity of MSC_S and MSC_M specimens was 7.85% and 7.30% under outdoor in situ exposure conditions, while that was 6.47% and 5.93%, respectively, under indoor conditions, as displayed in Figure 10a. This also confirms that the synergistic effect of silica fume and calcium sulfate whiskers has densified the microstructure of manufactured-sand concrete.

According to the previous results [46], the pore structure can be classified into four pore size ranges: harmless pores (<20 nm), slightly harmful pores (20–50 nm), harmful pores (50–200 nm), and highly harmful pores (>200 nm). On this basis, the pore volume distribution and related proportion can be obtained, as shown in Figure 10a and Figure 10b, respectively. Compared to the indoor testing condition, the volume of slightly harmful pores and harmful pores in the specimen under outdoor in situ exposure conditions has significantly increased. In detail, the total number of slightly harmful pores and harmful pores of MSC_S and MSC_M specimens was 0.0181 mL/g and 0.0193 mL/g under outdoor in situ exposure conditions, with 32.0% and 40.9% more than that under indoor testing conditions. Previous studies have shown that the evolution of these two types of pore structures is a crucial factor influencing the mechanical properties of cement-based materials [43,47]. This may be the reason why the mechanical properties and water resistance of manufactured-sand concrete exposed to high-altitude environments are even worse. In addition, it can be observed that the proportion of pores with a size of more than 200 nm in the MSC_M specimen has decreased, indicating that silica fume and calcium sulfate whiskers have refined the pore structure, thereby enhancing the performance of manufactured-sand concrete.

Drying shrinkage of cement-based materials is mainly related to the mesopores with a size of less than 50 nm, including gel pores and micro capillary pores [48]. The evaporation of free water in the plateau harsh environment causes the curvature of the liquid in the capillary pores to decrease, and the pore water in the small pores migrates to the larger pores under the action of capillary tension, increasing the drying shrinkage. Table 3 lists the mercury intrusion per unit of different pores. The mesopores (<50 nm) mercury intrusion of MSC_S and MSC_M specimens under outdoor testing conditions was 10.2% and 11.3% higher than that under indoor testing conditions, which can explain the reason for the increased drying shrinkage of manufactured-sand concrete in the plateau harsh environment. Meanwhile, the addition of silica fume, calcium sulfate whiskers and shrinkage-reducing agent results in a reduction in the mercury intrusion of gel pores and micro capillary pores, and thus reduces its drying shrinkage.

## 4. Discussion

**Figure 11:**
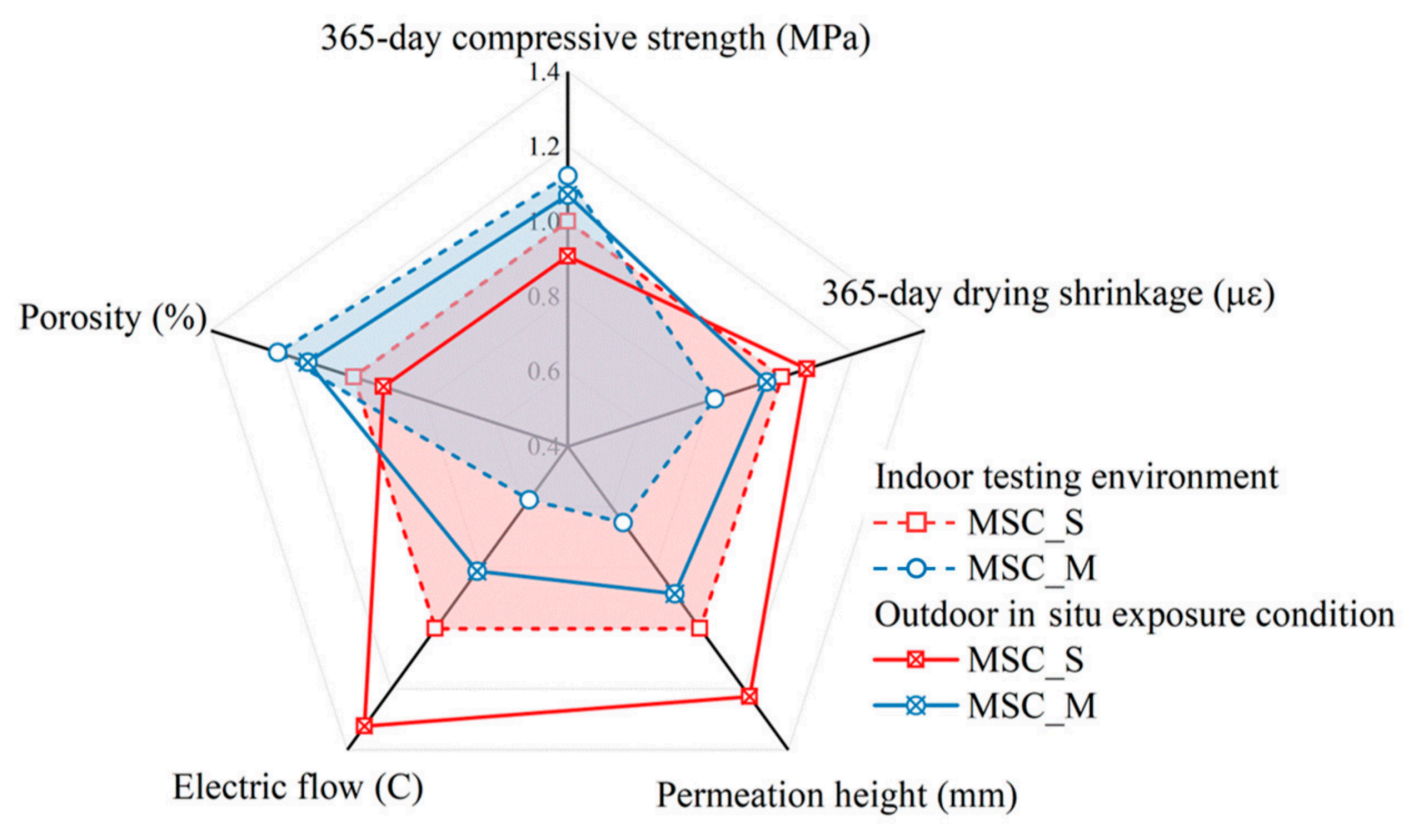
Comparison of long-term mechanical properties, drying shrinkage, and microstructure of manufactured-sand concrete.

As mentioned earlier, the plateau harsh environment does indeed have an impact on the long-term mechanical properties, drying shrinkage and creep behaviours of manufactured-sand concrete. This is mainly due to the deterioration of the microstructure, such as the pore structure coarsening and the water migration intensification. Here, based on the test data under standard conditions without adding any other materials, i.e., MSC_S specimen, the data for each condition are normalised, which can be defined as environmental impact coefficient, as shown in Figure 11. It can be observed that the influence of the plateau harsh environment on the macroscopic properties is relatively small, but that on the microscopic properties—such as, porosity, electric flow, and capillary water absorption—is large. This difference indicates that the plateau harsh environment causes specific damage to the microscopic structure [41,42]. Therefore, paying attention to the microstructure deterioration and its influencing factors is of great significance for clarifying the damage mechanisms related to the harsh environment at high altitudes.

The addition of silica fume and calcium sulfate whiskers makes the microstructure of manufactured-sand concrete denser, which is directly manifested by a significant reduction in the environmental impact coefficient, especially the porosity, electrical conductivity and water absorption height [35,36]. This phenomenon is attributed to the intensified moisture migration of concrete caused by the large temperature difference and low humidity in the plateau area, which leads to the coarsening of the pore structure and subsequently deteriorates its macroscopic properties.

It must be acknowledged that there are indeed better combinations than silica fume–calcium sulfate whiskers, such as nano-silica, graphene oxide, nano-calcium carbonate whiskers, etc. However, for large-scale projects like railways, when all indicators meet the design requirements, economic cost still needs to be one of the important factors to consider. Compared with materials like nano-silica, the economic cost increase in using silica fume–calcium sulfate whiskers is very small (only an increase of $ 10 per cubic metre of concrete [49]). Therefore, choosing the silica fume–calcium sulfate whiskers combination can effectively guide the actual construction engineering.

## 5. Conclusions

In this work, a 1-year measurement of the mechanical properties, drying shrinkage, and creep behaviour of manufactured-sand concrete in plateau regions was conducted at a test location in Xizang, China (32°52′ N, 97°06′ E, 3800 m altitude). The following conclusions can be obtained.

The comparative analysis in this work shows that the plateau harsh environment significantly deteriorates the long-term mechanical properties, drying shrinkage, and creep of manufactured-sand concrete. Compared to the indoor testing conditions (20 °C, 60% RH), the 365-day compressive strength and elastic modulus of manufactured-sand concrete under the plateau harsh environment dropped by at most 10.2% and 5.3%, respectively, while the 365-day drying shrinkage and specific creep increased by at most 15.3% and 9.4%, respectively. This is related to the coarsening pore structure caused by the harsh environment, and the pore volume ranging from 20 to 200 nm significantly increases.

The synergistic effect of silica fume, calcium sulfate whiskers and shrinkage-reducing agent can enhance the mechanical properties of manufactured-sand concrete in plateau harsh environments, and reduce its drying shrinkage and specific creep. Specifically, the 365-day compressive strength increased by 12.3%, while the drying shrinkage and specific creep were reduced by 10.5% and 16.6%, respectively. This is related to the dense microstructure effect of silica fume and calcium sulfate whiskers.

Overall, this study has identified the long-term performance evolution of manufactured-sand concrete in the plateau area, and has verified the improvement effects of silica fume and calcium sulfate whiskers on it. This is of great significance for guiding actual construction. However, this work mainly focuses on the macroscopic properties, and further analysis is required for the microstructure and phase composition. Meanwhile, this work was conducted only in one high-altitude area (32°52′ N, 97°06′ E), and its applicability in other high-altitude regions still needs further verification.

## Figures and Tables

**Table 1:** Chemical compositions of cementitious materials (wt %).

Raw Material	SiO_2_	Al_2_O_3_	CaO	Fe_2_O_3_	SO_3_	MgO	Na_2_O	LOI
Cement	21.3	4.72	63.7	3.12	2.06	2.61	0.59	1.55
Fly ash	40.7	20.94	5.52	5.24	1.59	1.36	1.10	4.72
Silica fume	96.2	0.31	0.32	0.05	1.26	0.26	0.93	0.47

**Table 2:** Mix proportion of manufactured-sand concrete (kg/m^3^).

No.	Cement	Fly Ash	MS	Coarse Aggregate	Silica Fume	CSW	Water	SP	SRA
MSC_S	428	76	706	1059	0	0	151	5.04	0
MSC_M	413	76	706	1059	10.08	5.04	151	2.52	2.52

Note: MS denotes manufactured sand, CSW is calcium sulphate whiskers, SP means superplasticiser, and SRA represents shrinkage-reducing agent.

**Table 3:** Mercury intrusion per unit of different pores.

Test Condition		Proportion of Pore Distribution					
		<50 nm		50–100 nm		>100 nm	
		%	mL/g	%	mL/g	%	mL/g
Indoor testing environment	MSC_S	56	0.0146	8	0.0022	35	0.0092
	MSC_M	62	0.0141	8	0.0018	30	0.0070
Outdoor in situ exposure condition	MSC_S	49	0.0161	15	0.0048	36	0.0117
	MSC_M	50	0.0157	18	0.0056	32	0.0101

## Data Availability

The original contributions presented in this study are included in the article. Further inquiries can be directed to the corresponding author.
